# Process validation and preclinical development of a new PET cerebral blood flow tracer [^11^C]MMP for initial clinical trials

**DOI:** 10.1186/s41181-024-00285-9

**Published:** 2024-07-23

**Authors:** Jun Toyohara, Tetsuro Tago, Muneyuki Sakata

**Affiliations:** Research Team for Neuroimaging, Tokyo Metropolitan Institute for Geriatrics and Gerontology, 35-2 Sakae- cho, Itabashi-ku, Tokyo, 173-0015 Japan

**Keywords:** Positron emission tomography, Cerebral blood flow, MMP, Carbon-11, Dosimetry, Toxicology

## Abstract

**Background:**

2-deoxy-2-[^18^F]fluoro-D-glucose ([^18^F]FDG) is commonly used for diagnosis of dementia because brain glucose metabolism reflects neuronal activity. However, as [^18^F]FDG is an analogue of glucose, accumulation of tracer in the brain is affected by plasma glucose levels. In contrast, cerebral blood flow (CBF) tracers are theoretically unaffected by plasma glucose levels and are therefore expected to be useful alternatives for the diagnosis of dementia in patients with diabetes. The techniques currently used for CBF imaging using single photon emission computed tomography (SPECT) and [^15^O]H_2_O positron emission tomography (PET), but these are limited by their insufficient resolution and sensitivity for regional brain imaging, especially in patients with brain atrophy. *N*-isopropyl-4-[^11^C]methylamphetamine ([^11^C]MMP) is a possible CBF tracer with high resolution and sensitivity that exhibits comparable performance to that of [^15^O]H_2_O in conscious monkey brains. We performed process validation of the radiosynthesis and preclinical development of [^11^C]MMP prior to clinical translation.

**Results:**

The decay-corrected yields of [^11^C]MMP at the end of synthesis were 41.4 ± 6.5%, with 99.7 ± 0.3% radiochemical purity, and 192.3 ± 22.5 MBq/nmol molar activity. All process validation batches complied with the product specifications. The acute toxicity of MMP was evaluated at a dose of 3.55 mg/kg body weight, which is 10,000 times the potential maximum clinical dose of [^11^C]MMP. The acute toxicity of [^11^C]MMP injection at 150 or 200 times, to administer a postulated dose of 740 MBq of [^11^C]MMP, was also evaluated after the decay-out of ^11^C. No acute toxicity of MMP and [^11^C]MMP injection was found. No mutagenic activity was observed for MMP. The effective dose calculated according to the Medical Internal Radiation Dose (MIRD) method was 5.4 µSv/MBq, and the maximum absorbed dose to the bladder wall was 57.6 µGy/MBq. MMP, a derivative of phenylalkylamine, showed binding to the sigma receptor, but had approximately 1/100 of the affinity of existing sigma receptor imaging agents. The affinity for other brain neuroreceptors was low.

**Conclusions:**

[^11^C]MMP shows acceptable pharmacological safety at the dose required for adequate PET imaging. The potential risk associated with [^11^C]MMP PET imaging is well within the acceptable dose limit.

**Supplementary Information:**

The online version contains supplementary material available at 10.1186/s41181-024-00285-9.

## Background

Most of the energy for brain activity is supported by the oxidative metabolism of glucose, but as the storage of oxidative substrates in brain tissue is extremely low, the brain requires a continuous supply of glucose and oxygen from the bloodstream. Therefore, in healthy brain tissues, neuronal activity, glucose consumption, and blood flow are well correlated. This is the basis for using 2-deoxy-2-[^18^F]fluoro-D-glucose ([^18^F]FDG), a sugar analogue, to study neuronal function in health and disease (Ishii 2002; Shimada et al. 2017). One of the most common applications of [^18^F]FDG in brain imaging is in the diagnosis of dementia, especially Alzheimer’s disease (AD) (Minoshima 2003). As [^18^F]FDG positron emission tomography (PET) enables clear anatomical and functional localization in the brain, considerable diagnostic information can be obtained for dementia (Minoshima et al. 1994, 2022). For example, AD is characterized by a prominent decrease in [^18^F]FDG uptake in the posterior cingulate, precuneus, and/or temporoparietal lobe, which is termed AD pattern. In contrast, in frontotemporal dementia, as the name suggests, decreased [^18^F]FDG uptake is observed in the frontal lobe and inferior temporal surface, whereas in dementia with Lewy bodies, the characteristic finding is decreased [^18^F]FDG uptake in the occipital lobe (Minoshima et al. 2022).

Although [^18^F]FDG is a standard tracer used for the clinical diagnosis of dementia, its diagnostic accuracy has been reported to be hampered in subjects with poorly controlled glycemia, for example in those with diabetes mellitus (DM) (Burns et al. 2013; Ishibashi et al. 2015). The findings of these previous studies imply that an individual with elevated plasma glucose levels can be mistakenly diagnosed with AD when [^18^F]FDG is used. Theoretically, [^18^F]FDG competes with glucose in glucose transporters and hexokinases (Wienhard 2002) and therefore affects the uptake of [^18^F]FDG in the brain.

As the prevalences of both dementia and DM inevitably increase with age (Cowie et al. 2010; Wada-Isoe et al. 2012), there is a rapid increase in patients with both DM and dementia in an ageing society. Therefore, an alternative to [^18^F]FDG is needed for the diagnosis of dementia in patients with DM. The most promising option is measurement of regional cerebral blood flow (CBF) (Matsuda et al. 2007; Tang et al. 2004) because neuronal activity is closely coupled to regional changes in CBF (Attwell and Iadecola 2002; Villringer and Dirnagl 1995). However, current modalities such as single photon emission computed tomography (SPECT) and [^15^O]H_2_O PET have limitations for the early diagnosis of dementia due to their low image resolution. As ageing causes atrophy evident as enlargement of the ventricles and dilation of the cerebral sulcus, low-resolution images can underestimate accumulation. To address this situation, we developed *N*-isopropyl-*p*-[^11^C]methylamphetamine ([^11^C]MMP) (Toyohara et al. 2020a) as a carbon-11 alternative to the standard SPECT CBF tracer *N*-isopropyl-*p*-[^123^I]iodoamphetamine (Ishii et al. 2009). [^11^C]MMP was highly incorporated and widely distributed in grey matter regions in the brains of conscious monkeys. The local distribution pattern of [^11^C]MMP closely resembled that of [^15^O]H_2_O, and the standardised uptake value (SUV) of the early phase scan showed a good correlation with the regional CBF calculated by [^15^O]H_2_O PET. These promising data prompted us to develop [^11^C]MMP as a novel CBF tracer for the diagnosis of dementia in patients with DM. Before entering the initial clinical trials, it is mandatory to perform a process validation of [^11^C]MMP production for clinical use and evaluate the preclinical toxicity and radiation dosimetry estimate from mouse biodistribution data.

## Methods

### General

The MMP was custom synthesized by the Nard Institute (Kobe, Japan) using methods described previously (Toyohara et al. 2020a). All other chemical reagents were obtained from commercial sources. Male ddY mice were obtained from Japan SLC (Hamamatsu, Japan). Sprague–Dawley rats [Crl: CD(SD)] were obtained from Jackson Laboratory Japan (Yokohama, Japan). Animal studies were approved by the Animal Care and Use Committee of the Tokyo Metropolitan Institute for Geriatrics and Gerontology (Approval Nos. 22,015 and 23,007) and Nihon Bioresearch (Hashima, Japan; Approval Nos. 430,085 and 430,086). Acute toxicity studies were performed under the “Revisions of the Guidelines for Single and Repeated Dose Toxicity Studies” (PMDA, 1993). Ames test was performed under the “Guidance on Genotoxicity Testing and Data Interpretation for Pharmaceuticals Intended for Human Use” (PMDA, 2012).

### *N*-isopropyl-1-(4-(4,4,5,5-tetramethyl-1,3,2-dioxaborolan-2-yl)phenyl)propan-2-amine

The pinacol boronic ester (Bpin) precursor, *N*-isopropyl-1-(4-(4,4,5,5-tetramethyl-1,3,2-dioxaborolan-2-yl)phenyl)propan-2-amine, was purchased from Aquila Pharmatech (Waterville, OH). Characterization of the Bpin precursor was performed by ^1^H nuclear magnetic resonance (NMR) and high-resolution electrospray ionization-mass spectrometry (HR-ESI-MS) analysis. ^1^H-NMR spectra were recorded in CDCl_3_ as a solution using tetramethylsilane as the internal standard on a JEOL 400 MHz spectrometer (Akishima, Japan). Multiplicities are indicated as s (singlet), d (doublet), or m (multiplet). HR-ESI-MS spectra were recorded on a Q Exactive equipped with an Ultimate 3000 high-performance liquid chromatography (HPLC) system (Thermo Fisher Scientific, Waltham, MA). ^1^H NMR (CDCl_3_, 400 MHz): δ 7.73 (d, *J* = 8.0 Hz, 2 H), 7.17 (d, *J* = 4.0 Hz, 2 H), 3.03 (m, 1H), 2.93 (m, 1H), 2.78 (m, 1H), 2.58 (m, 1H), 1.34 (s, 12 H), 1.01 (m, 9 H). HRMS (ESI) m/z calculated for C_18_H_30_BNO_2_ [M + H]^+^ 304.2554 found 304.2442. Chemical purity of the Bpin precursor was determined by HPLC analysis. For analysis, an analytical column [Inertsil ODS-3 5-µ, 4.6-mm inner diameter (id) × 250-mm length] purchased from GL Science (Kyoto, Japan) was used and an isocratic elution was applied using CH_3_CN/H_2_O/trifluoroacetic acid (45/55/0.1, v/v/v) at a flow rate of 1 mL/min [ultraviolet (UV) detector at 245 nm]. Retention time (rt) of the Bpin precursor was 5.0 min (purity, 94.7%).

### Automated radiosynthesis

**Fig. 1:**
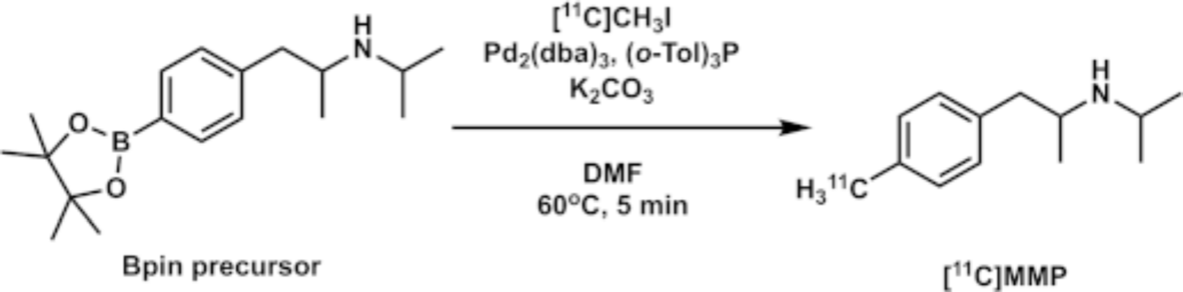
Radiosynthesis of [^11^C]MMP

[^11^C]MMP was synthesized by methylation of the Bpin precursor with [^11^C]CH_3_I in a palladium-promoted Suzuki cross-coupling reaction (Fig. 1) (Doi et al. 2009). The [^11^C]MMP labeling conditions were the same as those optimized for labeling phenylboronate reported previously (Table 1, entry 4 and scheme 3; Doi et al. 2009). The HPLC analytical yield of [^11^C]MMP under these conditions was greater than 90% (Additional file1: Fig. S2), and the reaction was nearly quantitative. Therefore, no further optimization of the conditions was performed.

**Table 1:** Product release specification and validation test results of [^11^C]MMP

Item	Release criteria^1^	Run 1	Run 2	Run 3
Manufacturing data				
Volume	15 ± 10 mL	15.6 mL	15.2 mL	15.2 mL
Radioactivity amount at end of synthesis	≥ 740 MBq	4960 MBq	7050 MBq	6520 MBq
Radioactivity concentration at end of synthesis	≥ 74 MBq/mL	318 MBq/mL	464 MBq/mL	429 MBq/mL
Estimated decay-corrected yield^2^	N/A	34.4%	47.3%	42.6%
Bubble point test (psi): NLT 40 psi	≥ 40 psi	50 psi	50 psi	50 psi
Quality control data				
Visual inspection	Clear, colourless to slightly yellow solution, free of particulate matter	Conforms	Conforms	Conforms
Radiochemical identity	Retention time of [^11^C]MMP peak within ± 15% min compared with the retention time of the known reference MMP peak	10.3%	8.6%	3.6%
Radionuclide identity by 511 keV peak	Peak energy of gamma ray spectrum at 511 keV	Conforms	Conforms	Conforms
Radionuclide identity by half-life determination	18.3–22.4 min	20.6 min	20.4 min	20.5 min
Content of ethanol	≤ 78.9 mg/mL	0.00 mg/mL	0.00 mg/mL	0.00 mg/mL
Residual acetonitrile	≤ 0.41 mg/mL	N.D.	N.D.	N.D.
Residual dimethylformamide	≤ 0.88 mg/mL	N.D.	N.D.	N.D.
Residual palladium	≤ 1 µg /mL	≤ 0.1 µg /mL^3^	≤ 0.1 µg /mL	≤ 0.1 µg /mL
pH	4.0–8.0	5.53	5.42	5.27
Radiochemical purity at release	≥ 90.0%	99.4%	99.9%	99.9%
Molar activity at end of synthesis	≥ 10 MBq/nmol	184.2 MBq/nmol	222.9 MBq/nmol	169.7 MBq/nmol
Bacterial endotoxin	< 150EU/vial	54.4 EU/vial	26.6 EU/vial	26.6 EU/vial
Sterility testing	No growth observed in 14 days	Conforms	Conforms	Conforms

^1^Release criteria of quality control data apply to a standard volume of 10 mL

^2^Based on [^11^C]CH_3_I.

^3^Detection limit is 0.1 µg/mL

[^11^C]CO_2_ was produced by proton irradiation of a gas mixture of 99.5% nitrogen/0.5% oxygen (Taiyo Nippon Sanso JFP, Kawasaki, Japan) at 50 µA for 30 min using an HM-20 cyclotron (Sumitomo Heavy Industries, Tokyo, Japan). [^11^C]CH_3_I was produced from [^11^C]CO_2_ with a CFN-MPS100 multipurpose synthesiser (Sumitomo Heavy Industries). A flow chart of the synthesizer is shown in Additional file 1: Fig. S1. A solution of tri(*o*-tolyl)phosphine (3.9 mg, 12.8 µmol) in *N*,*N*-dimethylformamide (DMF) (0.15 mL) and the Bpin precursor (2.4 mg, 8 µmol) in DMF (0.1 mL) was prepared. Immediately before the end of irradiation, this solution was added to a dry septum-equipped vial (Additional file 1: RV2 in Fig. S1) containing a mixture of K_2_CO_3_ (1 mg, 7.2 µmol) and tris(dibenzylideneacetone)dipalladium(0) (2.9 mg, 3.2 µmol). The [^11^C]CH_3_I produced was trapped in the reaction mixture of DMF (0.25 mL) with air cooling. The reaction mixture was heated to 60 °C for 5 min. After adding 1.5 mL of a dilute solution (CH_3_CN/25 mM CH_3_COOH/25mM CH_3_COONH_4_ = 15/42.5/42.5, v/v/v), the reaction mixture was passed through Fine Filter F (Forte Grow Medical, Sano, Japan) equipped with glass fiber wool, followed by injection onto the preparative HPLC: YMC-Pack Pro C18 RS S-5 μm [10-mm id × 250-mm length, YMC, Kyoto, Japan] with a mobile phase of CH_3_CN/50 mM CH_3_COOH/50mM CH_3_COONH_4_ (30/35/35, v/v/v) at a flow rate of 4.5 mL/min (UV detector at 228 nm). The rt of [^11^C]MMP was 6.5 min (Additional file 1: Fig. S2). The fraction of [^11^C]MMP was collected in a flask containing 0.2 mL of 250 mg/mL ascorbate injection (Nichi-Iko Pharmaceutical, Toyama, Japan) and 0.2 mL of 10% polyoxyethylene (20) sorbitan monooleate (polysorbate 80) (MP Biomedicals, Santa Ana, CA) in ethanol (Imazu Chemical, Tokyo, Japan) and evaporated to dryness. The residue was dissolved in 20 mL of physiological saline containing 0.125% (v/v) polysorbate 80, and the solution was filtered through a 0.22-µm membrane filter (Millex GV, Merck Millipore, Billerica, MA).

### Quality control

Filter integrity was assessed using a bubble point tester (SLTEST000; Merck Millipore). The pH value of the injection solution was determined using a pH meter (LAQUA F-72; Horiba, Kyoto, Japan). Residual solvents in the injection solution were measured by capillary gas chromatography using a GC-2014 or GC-2030 system with LabSolutions software (Shimadzu, Kyoto, Japan). Radionuclide was identified by determining half-life measurement using a Capintec CRC-55tR dose calibrator (Florham Park, NJ) and photopeak measurement at 511 keV using a NaI(Tl) radiation detector (US-2000, Universal Giken, Odawara, Japan). The limulus amoebocyte lysate (LAL) test was performed on a Toxinometer ET-6000 (Fujifilm Wako Pure Chemical, Osaka, Japan). Finally, a sample of the product formulation was tested for sterility post-release using direct inoculation in accordance with the Japanese Pharmacopeia, 18th edition (PMDA, 2021).

HPLC analysis was performed on a Shimadzu Prominence HPLC system equipped with a model LC-20AD pump, a model SPD-20A UV absorbance detector (set at 220 nm), a GABI 3 × 3-inch NaI scintillation detector (Elysia-Raytest, Straubenhardt, Germany), and an analytical column (Titan C18 1.9-µm, 2.1-mm id × 50-mm length) purchased from Sigma-Aldrich (St. Louis, MO). Operation of the Shimadzu Prominence HPLC system was controlled using Shimadzu LabSolutions software. For analysis, an isocratic elution was applied using CH_3_CN/50 mM CH_3_COOH/50mM CH_3_COONH_4_ (20/40/40, v/v/v) at a flow rate of 0.25 mL/min. The rt of the authentic standard MMP was 6.2 min (Additional file 1: Fig. S3).

The residual amounts of Pd in the final product were analysed with an inductivity coupled plasma mass spectrometer (ICP-MS) (Agilent 7700x; Agilent, Santa Clara, CA). The detection limit is approximately 100 ng/mL. ICP-MS analysis was performed at the Shimadzu Techno-Research (Kyoto, Japan).

### Toxicity study

In Japan, the structure of nonclinical toxicology studies for PET drugs in clinical research is the responsibility of the ethics committee of each institution or the subcommittee of specialities of PET drugs. According to the method used previously for approval of PET drugs for clinical use in our institute (Toyohara et al. 2013, 2016, 2020b; Sakata et al. 2017), we performed single-arm acute toxicology studies of the active pharmaceutical ingredient (MMP) and final dose formulation ([^11^C]MMP injection). Mutagenicity tests will also be conducted, as there are concerns about stochastic effects on genotoxicity.

Acute toxicity studies were performed at the Hashima Research Center, Nihon Bioresearch, under non-Good Laboratory Practice (GLP) control. Acute toxicity of MMP was tested in Sprague–Dawley rats. MMP at a dose of 3.55 mg/kg body weight (0.71 mg/mL in 10 v/v% dimethyl sulfoxide containing water for injection) was injected intraperitoneally into 5-week-old rats weighing 143–172 g (male, *n* = 5) and 129–135 g (female, *n* = 5). The dose of 3.55 mg/kg body weight is the 10,000-fold equivalent of the postulated maximum administration dose (1.85 nmol/0.355 µg/kg body weight) of 740 MBq [^11^C]MMP, with the lowest molar activity (Am) of 10 MBq/nmol for humans weighing 40 kg. Rats were observed frequently until 1 h and then at 2, 4, and 6 h after the injection on day 1, and thereafter once daily for 14 days for clinical signs of toxicity. Rats were weighed on days 1, 2, 4, 7, 10, and 14. At the end of the 14-day observation period, the rats were euthanized by exsanguination under isoflurane anaesthesia, and a macroscopic analysis of the autopsy samples was performed.

Three batches of [^11^C]MMP were prepared and tested after the decay-out of ^11^C. Solutions with decayed [^11^C]MMP were individually injected intravenously into 5-week-old male (162–174 g) and female (132–149 g) rats (*n* = 3 each) at doses of 4.05 µg/8.2 mL/kg body weight, 4.46 µg/7.5 mL/kg body weight and 5.86 µg/8.2 mL/kg body weight, for each of the three batches, equivalent to 150 and 200 times the postulated administration dose of 740 MBq [^11^C]MMP for humans. After injection of [^11^C]MMP, the rats were observed for clinical signs of toxicity for 14 days, and a macroscopic analysis was then performed as described above.

Mutagenicity tests were performed at the Hashima Research Center, Nihon Bioresearch, under non-GLP control. MMP was tested for mutagenicity by the Ames test with four histidine-requiring strains of *Salmonella typhimurium* (TA98, TA100, TA1535, and TA1537) and one strain of *Escherichia coli* (WP2*uvr*A), with and without the S9 mixture, at a dose range of 78.1–5000 µg/plate according to the standard method.

### Dosimetry

[^11^C]MMP (8.1 MBq/17 pmol) was injected intravenously into 8-week-old male ddY mice (37.8–42.4 g). The tracer-injected mice were housed individually in filter-paper-lined animal-rearing cages until the time of euthanasia. Mice were euthanized by cervical dislocation at 1, 5, 15, 30, 60, and 90 min after injection (*n* = 4 each). Blood was collected by heart puncture, and the tissues were harvested. Radioactivity excreted into urine was recovered from the cage floor and by cystocentesis from the urinary bladder. The samples were measured for ^11^C radioactivity with an auto-gamma counter (Hidex-AMG, Turku, Finland) and weighed. Tissue uptake of ^11^C was expressed as the percentage of injected dose per organ (%ID/organ) or the percentage of injected dose per gram of tissue (%ID/g). The tissue distribution data were extrapolated to an adult male phantom using the %kg/g method (Blau 1975). The radiation absorbed dose and effective dose for human adults were estimated using OLINDA/EXM software (Vanderbilt University, Nashville, TN) (Stabin et al. 2005). Effective dose was estimated using the risk-weighting factor of ICRP Publication 103 (ICRP 2007).

### Receptor selectivity

Interaction of MMP with 28 binding sites was examined, including major classes of neurotransmitter receptor, uptake system, and ion channels (Sekisui Medical, Naka, Japan). MMP was tested in all assays at 10 µM. Regarding the sigma receptor, a competitive inhibition curve was created for MMP and a positive control, haloperidol, and the concentration that inhibited binding by 50% (IC_50_ value) was calculated using GraphPad Prism ver. 10.2.3 (GraphPad Software, San Diego, CA). Each determination was made in duplicate.

## Results

### Automated radiosynthesis

The three batch production runs had activity yields of 6177 ± 1086 MBq, decay-corrected yields of 41.4 ± 6.5%, radiochemical purity of 99.7 ± 0.3%, and Am of 192.3 ± 22.5 MBq/nmol at the end of synthesis (Table 1). The average synthesis time following target bombardment was 32 min. All batches of [^11^C]MMP injection met the quality control (QC) criteria listed in Table 1. [^11^C]MMP was stable up to 90 min after end of synthesis, with acceptable appearance, pH of 5.36 ± 0.12, and radiochemical purity of 99.7 ± 0.2% (Table 2).

**Table 2:** Product specification and stability test results of [^11^C]MMP

Item	Criteria	Analysis time	Run 1	Run 2	Run 3
Visual inspection	Clear, colourless to slightly yellow solution, free of particulate matter	90 min	Conforms	Conforms	Conforms
Radiochemical identity	Retention time of [^11^C]MMP peak within ± 15% min compared with the retention time of the known reference MMP peak	30 min	10.2%	6.8%	4.1%
		60 min	9.9%	6.8%	3.0%
		90 min	10.1%	6.5%	4.3%
pH	4.0–8.0	30 min	5.46	5.39	5.23
		60 min	5.45	5.39	5.23
		90 min	5.46	5.39	5.23
Radiochemical purity	≥ 90.0%	30 min	99.8%	99.6%	99.8%
		60 min	99.8%	99.5%	99.7%
		90 min	99.9%	99.6%	99.6%

### Toxicity study

Acute toxicity in rats was evaluated after single intraperitoneal injection of MMP at a dose of 3.55 mg/kg and a single intravenous injection of one of the three batches of [^11^C]MMP preparation at a dose range of 4.05–5.86 µg/kg. There was no mortality in the rats during the 14-day observation period. All rat groups showed normal gains in body weight, and no clinical signs of toxicity were observed over the 15-day period. The post-mortem macroscopic examination found no abnormalities.

A reverse bacterial mutation test performed using *Salmonella thyphimurium* and *Escherichia coli* detected no mutagenic activity for MMP.

### Dosimetry

**Fig. 2:**
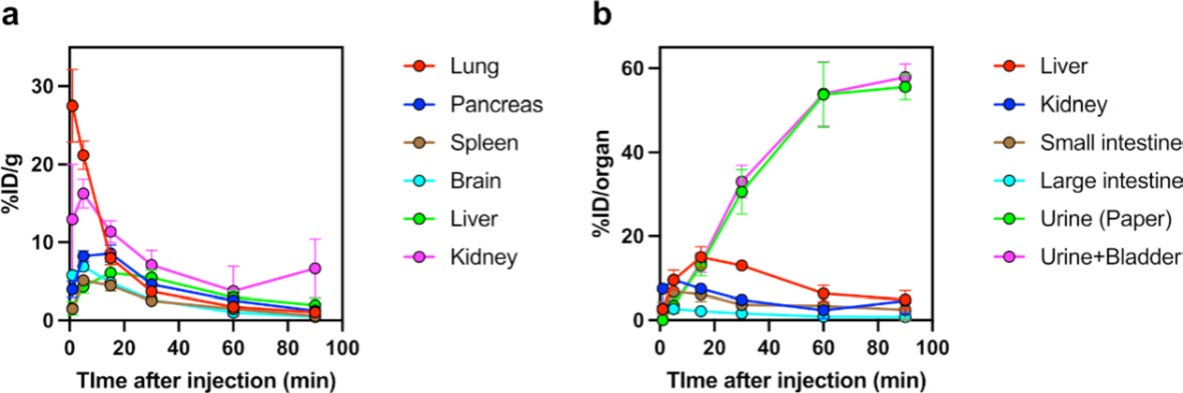
Regional decay-corrected time activity curves. High radioactivity concentration (**a**) and accumulation (**b**) are observed after intravenous injection of [^11^C]MMP into mice. Data are presented as the mean ± standard deviation (*n* = 4)

Tissue distributions of radioactivity after injection of [^11^C]MMP into mice are shown in Fig. 2, and the data are listed in Tables 3 and 4.

**Table 3:** Tissue distribution of radioactivity in mice after intravenous injection of [^11^C]MMP

	Injected dose/g tissue (%)*					
	1 min	5 min	15 min	30 min	60 min	90 min
Blood	1.40 ± 0.24	1.10 ± 0.14	0.72 ± 0.23	0.62 ± 0.21	0.56 ± 0.23	0.23 ± 0.09
Heart	8.23 ± 1.30	3.53 ± 0.21	1.93 ± 0.18	1.16 ± 0.22	0.56 ± 0.22	0.35 ± 0.14
Lung	27.53 ± 4.66	21.20 ± 1.81	7.97 ± 0.89	3.68 ± 0.50	1.73 ± 0.45	1.03 ± 0.13
Liver	1.56 ± 0.87	4.30 ± 0.84	6.07 ± 1.10	5.46 ± 0.97	2.93 ± 0.85	1.94 ± 0.88
Pancreas	3.96 ± 1.02	8.21 ± 0.70	8.55 ± 1.10	4.58 ± 0.52	2.52 ± 0.24	1.25 ± 0.34
Spleen	1.46 ± 0.42	5.09 ± 0.59	4.45 ± 0.69	2.48 ± 0.45	1.47 ± 0.44	0.56 ± 0.17
Kidney	12.93 ± 7.10	16.26 ± 1.85	11.36 ± 1.39	7.08 ± 1.91	3.73 ± 3.22	6.64 ± 3.76
Stomach	2.06 ± 1.30	3.24 ± 1.94	4.51 ± 1.35	2.69 ± 0.26	1.27 ± 0.89	1.52 ± 1.28
Small intestine	1.49 ± 0.84	3.65 ± 0.21	3.29 ± 0.83	2.14 ± 0.67	1.88 ± 0.59	1.34 ± 1.24
Large intestine	1.92 ± 0.95	2.36 ± 0.41	1.59 ± 0.62	1.22 ± 0.63	0.61 ± 0.27	0.61 ± 0.16
Testis	0.96 ± 0.24	1.26 ± 0.25	1.63 ± 0.16	1.69 ± 0.36	1.85 ± 0.35	1.47 ± 0.44
Muscle	1.77 ± 0.86	2.14 ± 0.14	1.22 ± 0.10	0.78 ± 0.17	0.51 ± 0.24	0.42 ± 0.18
Bone	2.21 ± 0.54	2.20 ± 0.30	1.29 ± 0.18	0.76 ± 0.26	0.49 ± 0.04	0.59 ± 0.14
Brain	5.75 ± 0.42	6.87 ± 0.62	4.83 ± 0.21	2.63 ± 0.22	1.01 ± 0.09	0.45 ± 0.08

*Mean ± standard deviation (*n* = 4)

**Table 4:** Organ distribution of radioactivity in mice after intravenous injection of [^11^C]MMP

	Injected dose/organ (%)*					
	1 min	5 min	15 min	30 min	60 min	90 min
Heart	1.56 ± 0.27	0.63 ± 0.05	0.33 ± 0.02	0.21 ± 0.01	0.11 ± 0.03	0.07 ± 0.03
Lung	5.54 ± 0.95	3.21 ± 0.65	1.47 ± 0.10	0.79 ± 0.09	0.36 ± 0.10	0.23 ± 0.03
Liver	2.63 ± 1.23	9.53 ± 2.41	14.98 ± 2.48	12.97 ± 1.05	6.29 ± 1.96	4.85 ± 2.18
Pancreas	0.76 ± 0.23	1.70 ± 0.23	1.74 ± 0.24	0.83 ± 0.03	0.47 ± 0.04	0.25 ± 0.05
Spleen	0.17 ± 0.07	0.57 ± 0.05	0.64 ± 0.12	0.35 ± 0.07	0.17 ± 0.02	0.08 ± 0.03
Kidney	7.44 ± 4.00	9.54 ± 0.98	7.47 ± 1.09	4.79 ± 1.06	2.37 ± 2.03	4.56 ± 2.94
Stomach	1.59 ± 1.06	1.88 ± 0.90	2.46 ± 0.69	1.76 ± 0.50	0.82 ± 0.44	0.84 ± 0.56
Small intestine	2.74 ± 1.46	6.77 ± 0.37	6.13 ± 1.74	3.66 ± 0.59	3.41 ± 0.77	2.47 ± 2.43
Large intestine	2.47 ± 1.19	2.68 ± 0.45	2.13 ± 1.04	1.59 ± 0.61	0.85 ± 0.51	0.77 ± 0.16
Testis	0.24 ± 0.03	0.32 ± 0.06	0.45 ± 0.05	0.52 ± 0.11	0.53 ± 0.15	0.49 ± 0.17
Brain	2.28 ± 0.25	2.68 ± 0.33	2.12 ± 0.06	1.09 ± 0.11	0.41 ± 0.06	0.21 ± 0.07
Bladder	0.07 ± 0.05	0.09 ± 0.03	0.60 ± 0.32	2.40 ± 1.59	0.23 ± 0.12	2.32 ± 1.75
Urine	0.00 ± 0.00	3.42 ± 1.82	13.03 ± 2.51	30.59 ± 5.33	53.71 ± 7.68	55.57 ± 3.04
Bladder + urine	0.07 ± 0.05	3.51 ± 1.80	13.64 ± 2.31	32.99 ± 3.90	53.94 ± 7.66	57.89 ± 3.18

*Mean ± standard deviation (*n* = 4)

Radioactivity concentrations in the blood decreased rapidly after [^11^C]MMP injection. The lung showed the highest initial radioactivity concentration (28%ID/g) and then rapidly decreased. The kidney showed the highest initial radioactivity (7.4%ID/organ) and peaked (9.5%ID/organ) at 5 min after injection and then slowly decreased. Excretion of radioactivity into the bladder and urine increased gradually in response to the clearance of radioactivity from the kidney, reaching 58%ID/organ (bladder + urine) at 90 min after injection. Radioactivity of the liver peaked (15%ID/organ) at 15 min after injection, before clearing. Radioactivity levels of the small intestine and large intestine (6.8%ID/organ and 2.7%ID/organ, respectively) peaked at 5 min, and showed clearance of radioactivity. These data suggest that radioactivity was excreted mainly by the urinary system.

The radiation absorbed dose was estimated from these biodistribution data (Table 5). The absorbed dose (µGy/MBq) was highest in the urinary bladder wall (57.6), kidney (8.6), lung (7.3), and pancreas (5.5). The effective dose according to the risk-weighting factors of ICRP publication 103 (ICRP 2007) was estimated as 5.36 µSv/MBq.

**Table 5:** Absorbed dose of [^11^C]MMP for human adults estimated from mouse data

Target organ	Absorbed dose (µGy/MBq)	Target organ	Absorbed dose (µGy/MBq)
Adrenal	2.21	Muscle	2.70
Brain	3.67	Ovaries	3.09
Breast	1.34	Pancreas	5.53
Gallbladder wall	2.43	Red marrow	2.12
Lower large intestine wall	4.03	Osteogenic cells	2.41
Small intestine	4.51	Skin	1.29
Stomach wall	3.59	Spleen	3.57
Upper large intestine wall	3.32	Testes	2.66
Heart wall	3.02	Thymus	1.70
Kidney	8.56	Thyroid	1.62
Liver	4.78	Urinary bladder wall	57.6
Lungs	7.29	Uterus	4.51
Total body	2.65		

Effective dose 5.36 µSv/MBq

### Receptor selectivity

MMP (10 µM) showed binding to the sigma receptor (inhibition 86%), but had approximately less than 1/100 of the affinity (IC_50_ = 1.1 µM) of the existing sigma receptor ligand, haloperidol (IC_50_ = 1.6 nM) (Additional file 1: Fig. S4). The affinity for other brain neuroreceptors was less activity (inhibition < 50%) (Additional file 1: Table S1).

## Discussion

We performed three process validation runs of [^11^C]MMP. The obtained activity yields (4960–7050 MBq) and high Am (169.7–222.9 MBq/nmol) were adequate for clinical research purposes. The three validation runs of [^11^C]MMP met the QC release criteria. Contamination of Pd in the final product was below the limit of detection (< 100 ng/mL). No degradation of [^11^C]MMP in formulation was confirmed up to 90 min. Taken together, the radiolabeling process of [^11^C]MMP on a CFN-MPS100 was of sufficient quality for clinical use.

In a previous study, [^11^C]MMP was synthesized by methylation of the corresponding tributyltin precursor with [^11^C]CH_3_I in a palladium-promoted Stille cross-coupling reactions. Although we have used Stille coupling for production of PET tracers for clinical use in our facility (Toyohara et al. 2009, 2011), anxiety exists regarding the contamination of toxic tin-containing by-products, which are difficult to remove perfectly from the reaction mixture. Palladium-mediated cross-coupling of boronic acid or boronic esters with electrophiles, termed Suzuki coupling, is an alternative to Stille coupling. In this study, we implemented a Bpin precursor and found that Suzuki coupling was more effective than Stille coupling using the corresponding tributyltin precursor. The isolated yield was significantly improved, by a factor of 3 to 4, compared to Stille coupling. Furthermore, the methylation reaction condition was milder in Suzuki coupling (60 °C for 5 min) than in Stille coupling (100 °C for 5 min). Another advantage of Suzuki coupling is that it does not use copper catalysts. A very small amount of copper (120 and 260 ng/mL) was detected in two samples of previous products (Toyohara et al. 2020a). Although this amount of Cu content in the final product was much lower than the limit set (34 µg/mL) as the permitted daily exposure in the “Guideline for Elemental Impurities [ICH-Q3D (R2)]” (PMDA, 2023), periodic analysis will be required to ensure the quality of the product.

The absence of any abnormality in rats in the acute toxicity test, together with the absence of mutagenicity of MMP, demonstrated the clinical suitability of [^11^C]MMP for first clinical trials in healthy volunteers. Taking into account the allometric scaling factor (Reagan-Shaw et al. 2008), the Non-Observed Adverse Effect Level (NOAEL) of MMP is estimated as > 3.0 µmol/0.58 mg/kg, which is 1,600 times the postulated maximum administration dose when 740 MBq of [^11^C]MMP with a low Am (10 MBq/nmol) is injected into a human subject of body weight 40 kg. Acute single intravenous administration toxicity test using decay-outed [^11^C]MMP final product, which includes all administered components, most reflects the clinical administration status. When considering the allometric scaling factor, NOAEL is estimated as at least 24.3 times the postulated maximum dose (740 MBq) of the tracer. As the calculated contents of MMP at the end of synthesis in three batch productions are ≤ 1.5 µg/740 MBq (0.7 ± 0.1 µg/740 MBq), the potential risk associated with [^11^C]MMP injection is considered to be within the toxicologically acceptable range (Koziorowski et al. 2017).

Radiation absorbed dose was highest in the urinary bladder wall, followed by kidney, lung, and pancreas. The effective dose was well within the previously reported range (3.2–14.1 µSv/MBq) for ^11^C-labelled PET tracers (Zanotti-Fregonara et al. 2021). In the case of administration of 740 MBq of [^11^C]MMP, effective dose was estimated as 4.0 mSv, which is within the strict limit of 10 mSv set by the ICRP recommendation (ICRP 1991) and practised in Europe. In this condition, the highest absorbed dose in the urinary bladder wall was estimated as 42.6 mGy, which is also within the strict limits for individual organs (30 mSv for sensitive organs and 50 mSv for all others), as required by the US Radioactive Drug Research Committee regulations (FDA, 2010).

MMP showed binding to the sigma receptor (IC_50_ = 1.1 µM). Since the estimated density of the sigma receptor in the human brain is approximately 3–60 nM, radioligands with nanomolar affinity (< 10 nM) are theoretically needed for imaging of the sigma receptor (Toyohara et al. 2021). Therefore, contribution of sigma receptors to the distribution of [^11^C]MMP in the brain is thought to be negligible.

## Conclusions

Automated synthesis of [^11^C]MMP for clinical use was achieved successfully and efficiently. All three batches of process validation complied with the product specifications. Preclinical toxicology studies indicated that [^11^C]MMP shows acceptable pharmacological safety at the dose required for adequate PET measurements. A single intravenous injection of 740 MBq of [^11^C]MMP leads to an estimated effective dose of 4.0 mSv and a highest absorbed dose to an organ of 42.6 mGy. The potential risk associated with [^11^C]MMP PET measurements is well within acceptable dose limits.

### Electronic supplementary material

Below is the link to the electronic supplementary material.

Supplementary Material 1

## Data Availability

The datasets used and/or analysed during the current study are available from the corresponding author on reasonable request.

## Abbreviations

AD: Alzheimer’s disease
Am: Molar activity
Bpin: Pinacol boronic ester
CBF: Cerebral blood flow
DM: Diabetes mellitus
DMF: N, N-dimethylformamide
FDA: US Food and Drug Administration
FDG: 2-deoxy-2-fluoro-D-glucose
GLP: Good laboratory practice
HPLC: High-performance liquid chromatography
HR-ESI-MS: High-resolution electrospray ionization-mass spectrometry
ICH: International Council for Harmonisation of Technical Requirements for Pharmaceuticals for Human Use
ICRP: International Commission on Radiological Protection
ID: Inner diameter
IMP: N-isopropyl-p-iodoamphetamine
LAL: Limulus amoebocyte lysate
MIRD: Medical internal radiation dose
NMR: Nuclear magnetic resonance
NOAEL: Non-observed adverse effect level
MMP: N-isopropyl-p-methylamphetamine
%ID: Percentage of injected dose
PET: Positron emission tomography
PMDA: Pharmaceuticals and Medical Devices Agency
Polysorbate 80: Polyoxyethylene (20) sorbitan monooleate
QC: Quality control
RDRC: Radioactive Drug Research Committee
RT: Retention time
SPECT: Single photon emission computed tomography
o-(Tol)_3_P: Tri(o-tolyl)phosphine
Pd_2_(dba)_3_: Tris(dibenzylideneacetone)dipalladium(0)
UV: Ultraviolet
