# Supplementary material for: Process validation and preclinical development of a new PET cerebral blood flow tracer [11C]MMP for initial clinical trials
Source: EJNMMI Radiopharm Chem. 2024 Jul 23;9:53. doi: 10.1186/s41181-024-00285-9 (PMC11266321; doi:10.1186/s41181-024-00285-9)
Supplement: Supplementary file 1 — Supplementary Material 1 [file 41181_2024_285_MOESM1_ESM.docx]

**Supplementary material**

**Fig. S1** Flow diagram of the CFN-MPS100 synthesizer for [^11^C]MMP production.

**Fig. S2** Representative semipreparative HPLC chromatogram of [^11^C]MMP. Radioactivity (**a**) and UV absorbance at 228 nm (**b**). HPLC solvent was changed to ethanol after elution of the product.

**Fig. S3** Representative analytical HPLC chromatogram of an [^11^C]MMP injection. Radioactivity (**a**) and UV absorbance at 220 nm (**b**). A large peak of ascorbic acid can be observed at the void in the UV chromatogram of [^11^C]MMP injection

**Fig. S4** Inhibition curves of test compound on binding of [^3^H]DTG to sigma receptor (non-selective). Data are expressed as mean values of duplicate samples.

**Table S1** Inhibition effect of MMP on radioligand binding to various receptors

| Assay system | Inhibition ratio (%) | | | Radioligand |
| --- | --- | --- | --- | --- |
|  | MMP | Positive substance | |  |
| Adenosine A_1_ | 3.26 | 98.97 | DPCPX | [^3^H]DPCPX |
| Adenosine A_2_ | 1.64 | 88.97 | NECA | [^3^H]CGS21680 |
| α_1_-Adrenergic (non-selective) | 44.47 | 100.0 | Prazosin | [^3^H]Prazosin |
| α_2_-Adrenergic (non-selective) | 42.47 | 89.82 | Yohimbine | [^3^H]Rauwolscine |
| β-Adrenergic (non-selective) | 32.80 | 97.25 | (±)-Propranolol | [^3^H]DHA |
| Dopamine D_1_ | 6.90 | 98.17 | *R*(+)-SCH-23390 | [^3^H]SCH23390 |
| Dopamine D_2_ | 6.04 | 95.06 | (+)-Butaclamol | [^3^H]Spiperone |
| GABA A (agonist site) | 3.62 | 96.50 | Muscimol | [^3^H]Muscimol |
| GABA A (BZ central) | 2.17 | 100.00 | Diazepam | [^3^H]Flunitrazepam |
| GABA B | 1.59 | 88.83 | GABA | [^3^H]GABA |
| Glutamate (non-selective) | 0.00 | 93.56 | L-Glutamic acid | [^3^H]Glutamic acid |
| Glutamate (AMPA) | 5.98 | 98.65 | (*S*)-AMPA | [^3^H]AMPA |
| Glutamate (Ketamine) | 11.86 | 97.79 | Kainic acid | [^3^H]Kainic acid |
| Glutamate (NMDA agonist site) | 4.63 | 100.00 | L-Glutamic acid | [^3^H]CGP-39653 |
| Glutamate (NMDA glycine site) | 0.22 | 97.35 | MDL105,519 | [^3^H]MDL105,519 |
| Glutamate (NMDA phencyclidine site) | 0.00 | 100.00 | (+)-MK-801 | [^3^H]MK-801 |
| Glycine (strychnine site) | 4.11 | 100.00 | Strychnine | [^3^H]Strychine |
| Histamine H_1_ (central) | 11.97 | 100.00 | Pyrilamine | [^3^H]Pyrilamine |
| Histamine H_2_ | 14.43 | 97.63 | Cimetidine | [^125^I]Iodoaminopotentidine |
| Histamine H_3_ | 3.82 | 99.25 | (*R*)(–)-α-Methylhistamine | [^3^H]N-methyl-histamine |
| Muscarinic M_1_ | 27.81 | 99.78 | Atropine | [^3^H]Pirenzepine |
| Muscarinic M_2_ | 23.38 | 99.84 | Atropine | [^3^H]AF-DX384 |
| Nicotinic (neuronal) | 0.00 | 95.62 | (±)-Nicotine | [^3^H]Cytisine |
| Opiate (non-selective) | 4.92 | 96.53 | Naloxone | [^3^H]Naloxone |
| Serotonin 5HT_1A_ | 15.84 | 95.99 | Serotonin | [^3^H]8-OH DPAT |
| Serotonin 5HT_2A_ | 24.12 | 98.94 | Ketanserin | [^3^H]Ketanserin |
| Serotonin 5HT_3_ (human) | 0.91 | 99.78 | Tropisetron | [^3^H]GR65630 |
| Sigma (non-selective) | 85.89 | 98.42 | Haloperidol | [^3^H]DTG |

Test substance concentration : 10 μM, positive substance concentration : 10 μM

Data are expressed as the mean values of duplicate samples.

The % inhibition was calculated from “100 – binding ratio”.

Binding ratio: [(B - N) / (B_0_ - N)] × 100 (%)

B: Bound radioactivity in the presence of test substance and positive substance (individual value)

B_0_: Total bound radioactivity in the absence of test substance and positive substance (mean value)

N: Non-specific bound radioactivity (mean value)
